# Correction: The Epstein–Barr virus nuclear antigen-1 upregulates the cellular antioxidant defense to enable B-cell growth transformation and immortalization

**DOI:** 10.1038/s41388-019-1066-1

**Published:** 2019-10-24

**Authors:** Jiayu Wang, Noemi Nagy, Maria G. Masucci

**Affiliations:** Department of Cell and Molecular Biology, Karolinska Institutet, Stockholm, Sweden

**Keywords:** Mechanisms of disease, Tumour virus infections

**Correction to: Oncogene 38**

10.1038/s41388-019-1003-3

This article was originally published under Springer Nature’s License to Publish, but has now been made available under a CC BY 4.0 license. The PDF and HTML versions of the paper have been modified accordingly.

